# Supplementary material for: Causal Relationship between Aging and Anorexia Nervosa: A White-Matter-Microstructure-Mediated Mendelian Randomization Analysis
Source: Biomedicines. 2024 Aug 16;12(8):1874. doi: 10.3390/biomedicines12081874 (PMC11351342; doi:10.3390/biomedicines12081874)
Supplement: Supplementary file 1 [file biomedicines-12-01874-s001.zip › Supplementary Figure/Figure S7/Single SNP plot of MR analysis for ubm-b-1631 to AN.pdf]

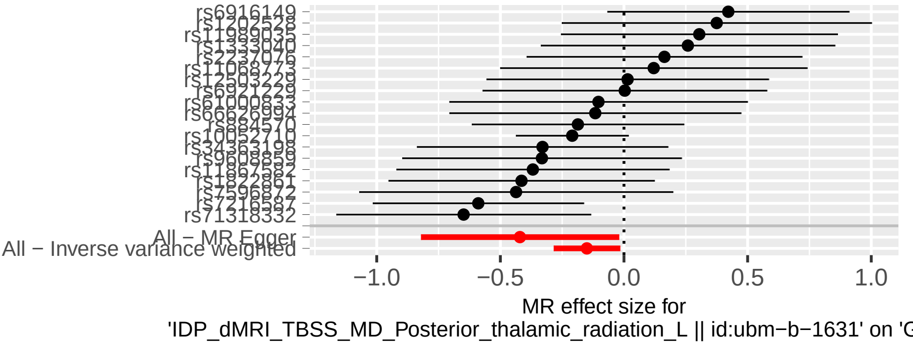
rs6916149
rs1202528
rs11989035
rs1333040
rs2237076
rs11068773
rs12503229
rs6921229
rs61000833
rs66626994
rs884570
rs10052710
rs34363198
rs9608859
rs11867582
rs1822861
rs7596872
rs7216587
rs71318332
All − MR Egger
All − Inverse variance weighted
−1.0
−0.5
0.0
0.5
1.0
MR effect size for
'IDP_dMRI_TBSS_MD_Posterior_thalamic_radiation_L || id:ubm−b−1631' on 'G
